# Prevalence, coinfection, and risk factors associated with *Fasciola hepatica* and other gastrointestinal parasites in cattle from the Peruvian Amazon

**DOI:** 10.14202/vetworld.2023.546-553

**Published:** 2023-03-22

**Authors:** Hugo Frias, César Maraví, Miguel A. Arista-Ruiz, Danitza I. Yari-Briones, Juan R. Paredes-Valderrama, Yesica Rojas Bravo, J. V. Cortez, G. T. Segura, Ricardo Encina Ruiz, Rainer M. Lopez Lapa, Nilton Luis Murga Valderrama

**Affiliations:** 1 Academic Department of Zootechnics, Faculty of Zootechnical Engineering, Agribusiness and Biotechnology, Universidad Nacional Toribio Rodríguez de Mendoza, Amazonas, Perú; 2 Laboratory of Infectious and Parasitic Diseases of Domestic Animals, Universidad Nacional Toribio Rodríguez de Mendoza, Amazonas, Perú; 3 Livestock and Biotechnology Research Institute, Universidad Nacional Toribio Rodríguez de Mendoza, Amazonas, Perú; 4 Research Unit, Instituto de Educación Superior Tecnológico Público Mache, La Libertad, Perú; 5 Department of Science School Veterinary Science, The University of Sydney, Sydney, Australia

**Keywords:** cattle, *Eimeria* spp, *Fasciola hepatica*, gastrointestinal parasites, prevalence, risk factors

## Abstract

**Background and Aim:**

Extensive cattle rearing is a major source of economy for the inhabitants of the Amazon region of Peru. Milk and meat production is generally affected by the prevalence of various parasites, including hepatic and gastrointestinal parasites, as these products provide ideal conditions for parasitic growth. This poses a serious public health threat. This study aimed to estimate the prevalence, coinfection, and risk factors associated with the liver fluke (*Fasciola hepatica)* and other gastrointestinal parasites in cattle from the Amazon region of Peru.

**Materials and Methods:**

Fecal samples obtained from 1450 bovine specimens were analyzed using flotation and sedimentation methods to identify parasites, including *Eimeria* spp., strongyle-type eggs (STEs), and *F. hepatica*. We collected information about the specimens, including age, sex, origin, breed, category, frequency of deworming, farm size, herd size, water sources, and rearing system by conducting simple inspections and interviewing owners. The data obtained were statistically evaluated using the Chi-square test (p < 0.05) to determine the association between the qualitative variables. We also calculated the odds ratio at a 95% confidence interval to identify the risk factors.

**Results:**

We observed that *F. hepatica*, *Eimeria* spp., and STEs were 45.6%, 39.8%, and 35.3% prevalent, respectively. We found risk factors related to distomatosis in the animals from Huambo, where the drinking water sources are mainly streams, ditches, and rivers, while the specimens from Valle Chico were predisposed to coccidiosis. Further, the risk factors related to the presence of STEs in feces were age (61–90 months), origin (Valle Chico), herd size (<50 animals), and type of extensive rearing. Furthermore, significant coinfection was observed between *Eimeria* spp. and STEs.

**Conclusion:**

The high percentages of parasites in cattle observed were related to epidemiological factors, such as the origin of the sample, water sources, age, herd size, and extensive breeding. Similarly, the presence of STEs was a risk factor for contracting coccidiosis. Our future goals include investigating these parasites using a larger sample size and identifying more risk factors using more sensitive and specific diagnostic tests.

## Introduction

Understanding parasitosis in domestic animals is crucial as it causes severe economic losses and threatens animal welfare and public health. Moreover, breeders are challenged with parasites resistant to anti-parasitic agents [1, 2]. Further, low cattle production due to infections by parasites, such as *Eimeria* spp. or other gastrointestinal helminths, is often unnoticed due to the subclinical symptoms found in most cattle [3, 4]. In addition, animals with high parasite loads contaminate pastures or water sources with parasite eggs, which enable the continual progression of the parasite’s biological cycle under optimal environmental conditions [5]. *Fasciola hepatica*, a highly studied parasite, uses cattle liver as its intermediate host. Therefore, this parasite is found in livestock farms worldwide [6] and directly impacts the economy and public health [7, 8].

Although parasites are highly prevalent in cattle and recorded in all countries, the associated risk factors are unclear and need to be investigated for their control and treatment. This will also improve public health, especially in areas endemic to *F. hepatica* or where cattle are raised.

This study aimed to estimate the prevalence and risk factors for *F. hepatica, Eimeria* spp., and other gastrointestinal parasites in cattle and the possible coinfections.

## Materials and Methods

### Ethical approval

During the present investigation, the health and integrity of all the animals involved in this study were safeguarded, especially those pregnant, geriatric, and neonatal animals. This project was approved by the Ethics Committee of the Universidad Nacional Toribio Rodríguez de Mendoza in the city of Chachapoyas, Peru (Approval no. CIEI-N° 013).

### Study period and location

**Figure-1:**
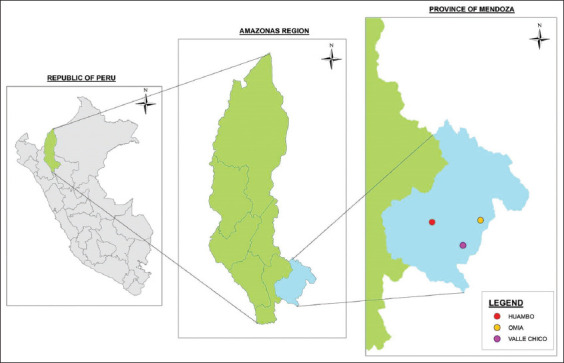
The map shows three towns in the Rodriguez de Mendoza Province of the Amazonas Region, northern Peru [Source: Map created using QGIS version 3.10}.

The study was conducted from November 2020 to February 2021 and involved the towns of Omia, Huambo and Valle Chico, belonging to the Rodríguez de Mendoza Province, Amazonas Region, located in northern Peru (Figure-1). The sampled cities have an average ambient temperature and relative humidity (RH) of 20°C and 75% RH, respectively, and with an annual rainfall of 876 mm.

### Animals and study variables

Fecal samples were collected from 1450 bovines and analyzed without anthelmintic treatment 3 months before sample collection to identify parasites, including *F. hepatica, Eimeria* spp., and strongyle-type eggs (STEs). We also compiled an epidemiological file filled out by the owners, including the variables such as age, sex, origin, breed, category, frequency of deworming, farm size, herd size, and water sources for drinking and rearing systems (Table-1).

**Table-1:** Frequency distribution of *Fasciola hepatica*, *Eimeria* spp. and STEs in bovines of the Amazon Region, associated with each study variable.

Study variable	*Fasciola hepatica*				*Eimeria* spp*.*				STEs			
		
	Positive		Negative		Positive		Negative		Positive		Negative	
					
	n	%	n	%	n	%	n	%	n	%	n	%
Age (months)												
0–30	323	43.2	425	56.8	286	38.2	642	61.8	255	34.1	493	65.9
31–60	301	48.5	320	51.5	254	40.9	367	59.1	216	34.8	405	65.2
61–90	36	46.2	42	53.8	35	44.9	43	55.1	38	48.7	40	51.3
>90	1	33.3	2	66.7	2	66.7	1	33.3	3	100.0	0	0.0
Sex												
Male	255	44.8	314	55.2	228	40.1	341	59.9	185	32.5	384	67.5
Female	406	46.1	475	53.9	349	39.6	532	60.4	327	37.1	554	62.9
Origin												
Omia	147	40.1	220	59.9	122	33.2	245	66.8	74	20.2	293	79.8
Huambo	243	50.4	239	49.6	176	36.5	306	63.5	110	22.8	372	77.2
Valle chico	271	45.1	330	54.9	279	46.4	322	53.6	328	54.6	273	45.4
Race												
Brown Swiss	239	50.3	236	49.7	189	39.8	286	60.2	168	35.4	307	64.6
Holstein	44	41.5	62	58.5	49	46.2	57	53.8	37	34.9	69	65.1
Simmental	200	41.0	288	59.0	194	39.8	294	60.2	158	32.4	330	67.6
Criole	178	46.7	203	53.3	145	38.1	236	61.9	149	39.1	232	60.9
Category												
Calf	86	45.3	104	54.7	67	35.3	123	64.7	51	26.8	139	73.2
Young bull	80	47.6	88	52.4	66	39.3	102	60.7	54	32.1	114	67.9
Bull	97	44.3	122	55.7	99	45.2	120	54.8	88	40.2	131	59.8
Heifer	76	42.5	103	57.5	77	43.0	102	57.0	74	41.3	105	58.7
Old heifer	50	43.5	65	56.5	45	39.1	70	60.9	40	34.8	75	65.2
Young cow	26	38.2	42	61.8	25	36.8	43	63.2	26	38.2	42	61.8
Cow	246	48.1	265	51.9	198	38.7	313	61.3	179	35.0	332	65.0
Frequent desparasitation												
Yes	227	45.2	275	54.8	221	44.0	281	56.0	203	40.4	299	59.6
No	434	45.8	514	54.2	356	37.6	592	62.4	309	32.6	639	67.4
Farm size												
Big	10	41.7	14	58.3	8	33.3	16	66.7	07	29.2	17	70.8
Medium	136	50.9	131	49.1	98	36.7	169	63.3	60	22.5	207	77.5
Small	515	44.4	644	55.6	471	40.6	688	59.4	445	38.4	714	61.6
Herd size												
51–100	139	48.3	149	51.7	101	35.1	187	64.9	80	27.8	208	72.2
1–50	522	44.9	640	55.1	476	41.0	686	59.0	432	37.2	730	62.8
Water resource												
Fountain	105	35.8	188	64.2	127	43.3	166	56.7	117	39.9	176	60.1
Ravine	358	46.7	409	53.3	286	37.3	481	62.7	248	32.3	519	67.7
Stream	68	49.3	70	50.7	53	38.4	85	61.6	32	23.2	106	76.8
Well	22	40.7	32	59.3	23	42.6	31	57.4	25	46.3	29	53.7
River	108	54.5	90	45.5	88	44.4	110	55.6	90	45.5	108	54.5
Bredding system												
Intensive	26	38.8	41	61.2	19	28.4	48	71.6	15	22.4	52	77.6
Extensive	634	45.9	748	54.1	557	40.3	825	59.7	497	36.0	885	64.0
Mixed	1	100.0	0	0.0	1	100.0	0	0	0	0.0	1	100.0

STEs=Strongyle-type eggs

### Sample collection and laboratory analysis

At least 100 g of fecal sample was manually collected from the bovine rectum in the morning and analyzed on the same day. The polyethylene gloves used for this procedure were labeled with the code on the respective epidemiological record. The samples were transported in expanded polystyrene boxes and sent to the Laboratory of Infectious and Parasitic Diseases of Domestic Animals, Livestock and Biotechnology Research Institute, Toribio Rodríguez, National University of Mendoza.

We utilized the flotation method to determine the presence of eggs and oocysts of gastrointestinal parasites. For this, 3 g of feces were ground in a mortar with distilled water, filtered through a fine mesh into plastic tubes, and centrifuged at 126× *g* for 3 min. The supernatant was removed, and 25 mL of the saturated sugar solution was added and incubated for 10 min until a convex meniscus formed at the edge. A cover slip was placed on the meniscus and kept on a slide for microscopic observation (Olympus, Japan, Model: BX53) [9].

As previously described by Dennis *et a*l. [10], the modified sedimentation test was used to detect *F. hepatica*, using 10 g of feces and 50 mL of detergent solution (Marsella). This mixture was washed repeatedly until a transparent supernatant was formed, and then, three drops of Lugol were added. Finally, the solution was poured into Petri dishes for microscopic observation at 20× and 40× magnification [9]. The samples with oocysts or STEs were considered positive for *Eimeria* spp. or STEs, respectively, while the presence of operculate, ellipsoidal, and yellowish-brown eggs indicated *F. hepatica*.

### Statistical analysis

The prevalence of *F. hepatica*, *Eimeria* spp., and STEs in the feces was expressed as percentages based on the data analysis. The association between qualitative variables was analyzed using the non-parametric Chi-square test, considering a significance value of p < 0.05. We also calculated the odds ratio (OR) and confidence interval (CI) with 95% reliability. The risk factors associated with the studied parasites were considered valid when OR and CI >1 and p < 0.05. The data were analyzed using IBM Statistical Package for the Social Sciences v.25 (IBM Corp., NY, USA).

## Results

**Figure-2:**
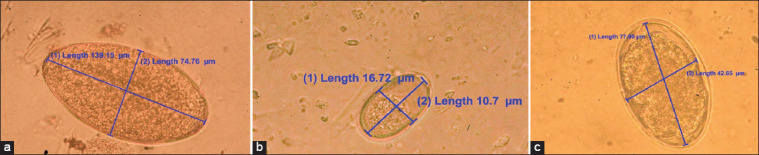
Parasite eggs observed under the light microscope in bovine fecal samples. (a) *Fasciola hepatica*, (b) *Eimeria* spp, (c) Strongyle-type eggs.

The morphology of the parasites was visualized using an optical microscope at 40× (*F. hepatica* and STE) and 20× (*Eimeria* spp.) (Figure-2) magnification. The overall prevalence of *F. hepatica* was 45.6% (95% CI; 43.1%–48.1%) and was statistically associated with variables, including origin, race, and water sources (Table-2). The risk factors associated with *F. hepatica* (OR: 1.52; CI 1.15–2.01; p < 0.01) also included the animals from Huambo, where the water sources included streams (OR: 1.56; CI: 1.18–2.07; p < 0.001), ditches (OR: 1.73; CI: 1.15–2.62; p < 0.001), and rivers (OR: 2.14; CI: 1.48–3.11 p < 0.001) (Table-2).

**Table-2:** Risk factors associated with infestation by *Fasciola hepatica*, *Eimeria* spp. and STEs in bovines of the Amazon Region, associated with each study variable.

Study variable	*Fasciola hepatica*			*Eimeria* spp.			STEs		
		
	OR	CI (95%)	p-value*	OR	CI (95%)	p-value*	OR	CI (95%)	p-value*
Global prevalence	45.6% (CI 95%: 43.1%–48.1%)			39.8% (CI 95%: 37.3%–42.3%)			35.3% (CI 95%: 32.3%–37.8%)		
Age (months)									
0–30	Ref	Ref	0.260	Ref	Ref	0.420	Ref	Ref	0.007
31–60	1.24	0.99–1.53		1.55	1.26–1.92		1.03	0.82–1.28	
61–90	1.13	0.71–1.80		1.82	1.14–2.92		1.83	1.14–2.93	
>90	0.65	0.06–7.28		4.48	0.41–49.71		----	----	
Sex									
Male	Ref	Ref	0.636	Ref	Ref	0.862	Ref	Ref	0.073
Female	1.05	0.85–1.31	0.98	0.79–1.21	1.22	0.98–1.53
Origin									
Omia	Ref	Ref	0.010	Ref	Ref	0.001	Ref	Ref	0.001
Huambo	1.52	1.15–2.01		1.15	0.86–1.53		1.17	0.83–1.63	
Valle chico	1.22	0.94–1.59		1.74	1.32–2.27		4.75	3.51–6.43	
Race									
Brown Swiss	Ref	Ref	0.025	Ref	Ref	0.511	Ref	Ref	0.236
Holstein	0.71	0.46–1.07		1.30	0.85–1.98		0.97	0.63–1.52	
Simmental	0.68	0.53–0.88		0.99	0.78–1.29		0.87	0.66–1.14	
Criole	0.86	0.66–1.13		0.92	0.71–1.23		1.17	0.88–1.55	
Category									
Calf	Ref	Ref	0.653	Ref	Ref	0.468	Ref	Ref	0.059
Young bull	1.09	0.73–1.67		1.18	0.77–1.82		1.29	0.81–2.03	
Bull	0.96	0.65–1.42		1.51	1.02–2.26		1.83	1.20–2.78	
Heifer	0.89	0.59–1.34		1.38	0.91–2.11		1.92	1.24–2.97	
Old heifer	0.93	0.58–1.48		1.18	0.73–1.90		1.45	0.88–2.39	
Young cow	0.74	0.42–1.31		1.06	0.60–1.89		1.68	0.93–3.02	
Cow	1.12	0.80–1.57		1.16	0.82–1.64		1.46	1.02–2.12	
Frequent desparasitation									
Yes	Ref	Ref	0.838	Ref	Ref	0.017	Ref	Ref	0.003
No	1.03	0.82–1.27	0.76	0.61–0.95	0.71	0.56–0.89
Farm size									
Big	Ref	Ref	0.146	Ref	Ref	0.401	Ref	Ref	0.001
Medium	1.45	0.62–3.38		1.15	0.47–2.81		0.70	0.27–1.77	
Small	1.11	0.49–2.54		1.36	0.58–3.22		1.51	0.62–3.67	
Herd size									
51–100	Ref	Ref	0.308	Ref	Ref	0.067	Ref	Ref	0.003
1–50	0.87	0.67–1.13	1.28	0.98–1.68	1.53	1.16–2.04
Water resource									
Fountain	Ref	Ref	0.001	Ref	Ref	0.229	Ref	Ref	0.001
Ravine	1.56	1.18–2.07		0.77	0.59–1.02		0.71	0.54–0.94	
Stream	1.73	1.15–2.62		0.81	0.53–1.23		0.45	0.28–0.71	
Well	1.23	0.68–2.22		0.96	0.53–1.74		1.29	0.72–2.32	
River	2.14	1.48–3.11		1.04	0.73–1.50		1.25	0.87–1.81	
Bredding system									
Intensive	Ref	Ref	0.289	Ref	Ref	0.070	Ref	Ref	0.048
Extensive	1.33	0.81–2.21		1.71	0.99–2.93		1.94	1.08–3.49	
Mixed	----	----		----	----		----	----	

Ref=Reference, OR=Odds ratio, CI 95%=Confidence interval at 95%,

* Chi-square non-parametric test

Table-2 shows that coccidiosis was 39.8% (95% CI: 37.3%–42.3%) prevalent and was significantly associated with the prevalence of *Eimeria* spp. and animals from the town of Valle Chico were risk factors (OR: 1.74; CI: 1.32–2.27; p < 0.05). The deworming frequency was also statistically associated with this parasite (Table-2).

Similarly, the total prevalence of STEs was 35.3% (95% CI: 32.3%–37.8%), which was significantly positively associated with variables, including age, origin, frequency of deworming, farm size, herd size, water sources, and rearing system. The cattle between 61 and 90 months of age (OR: 1.83; CI: 1.14–2.93; p < 0.05) and from the Valle Chico District (OR: 4.75; CI: 3.51–6.43; p < 0.05) were also considered as risk factors. Similar associations were seen in herds with <50 animals (OR: 1.53; CI: 1.16–2.04; p < 0.05) and extensive rearing (OR: 1.94; CI: 1.08–3.49; p < 0.05) (Table-2).

Overall, 229 animals were coinfected with *Eimeris* spp. and STEs, indicating a clear association between these two parasites (OR: 1.37; CI 95%: 1.02–1.71; p < 0.05). Although the highest percentage of animals were coinfected with *Eimeria* spp. and *F. hepatica*, we did not observe any significant or lower risk (Table-3).

**Table-3:** Percentage of cattle with parasite coinfection.

Coinfection	n	%	OR (CI 95%)	p-value*
*Eimeria* spp. *+ Fasciola hepatica*	259	17.86	0.95 (0.77–1.17)	0.664
*Eimeria* spp*. +* STE	229	15.79	1.37 (1.11–1.71)	0.005
*Fasciola hepatica + S*TE	222	15.31	0.87 (0.71–1.08)	0.208

OR=Odds ratio, CI 95%=Confidence interval at 95%, STEs=Strongly-type eggs,

* Non-parametric Chi-square test

## Discussion

Hepatic fluke infection is a significant public health concern that affects the economy and has been widely investigated. Parasitosis, directly and indirectly, affects cattle, resulting in changes in the intestinal microbiota and high morbidity, which reduce their productivity [11, 12]. Coccidiosis and other gastrointestinal parasitic infections are also relevant to animal health and production due to their epidemiological characteristics, resistance to humidity, survival of oocysts in feces, and high parasite loads, making them over 95% prevalent [13, 14].

Conditions, such as the year of sample collection, temperature, humidity, mowing of pastures, type of management on the farms, and the diagnostic test used directly influence the prevalence results for *F. hepatica* [15, 16]. A similar report showed a 42.3% (58/137) prevalence of *F. hepatica* in the Arequipa Region (Huanca district, Southern Peru), where the environmental conditions, including rainfall and RH, are lower than our study area [17]. Conversely, La Libertad (Pataz), located in northern Peru, has similar RH as our region, and the prevalence was 62.4% [18]. Therefore, considering these studies, we used coprological tests. The diagnostic tests used to identify the animals infested with *F. hepatica* showed divergent results. For example, another study evaluated the infested livers, which is a gold standard diagnostic test, in other provinces of the Amazon region, such as Bongará (93.9%), Chachapoyas (89.74%), and Luya (87.5%) and showed high percentage prevalence [19]. These conflicting reports result in misinterpretation of the actual prevalence as the animals that tested negative in the coprological tests could be positive in other tests. Moreover, lower percentages are present in the Colombian Sierra, which has similar humidity and temperature as the Amazon region. Here, the prevalence reached 22.3% [20], which is lower than that observed in the Amazon. This data highlights the importance of simultaneously applying other tests to measure the prevalence in real time.

In our study, the animal’s age was not identified as a risk factor for contracting liver fluke disease. This is contrary to the reports from European countries, including Denmark, where the highest risk was shown in heifers and cows [21]. Interestingly, we observed no significant association between the intensive and extensive exploitation system or herd size, as reported in another study in Ireland [22]. The water source (ravines, ditches, and rivers) variable presented a significantly high risk, which is strongly supported by studies explaining how the parasites need thin films of water to complete their biological cycle, in addition to intermediate host and humid environments [23, 24].

The divergent results regarding the prevalence and risk factors for coccidiosis are related to various epidemiological elements, such as environmental temperature, presence of flooded areas in the pastures, and management activities within the farm, including frequent emptying of drinkers or raising sheep together with cattle [25, 26]. The comparative reports regarding the prevalence of coccidiosis in cattle in Peru are limited due to the lack of information published in indexed journals. Moreover, conflicting results are observed worldwide. For example, coccidiosis has a 51% prevalence in Brazil, of which 71% of the positive results might be coinfested with two or more species of coccidia [27]. Colombia showed a prevalence of 75.5%, associated with variables such as soil type, feeding system, drinking system, and herd size [28]. Another study conducted in Colombia showed a prevalence of 19.4% [29], confirming that there may be considerable differences in the percentage of infested animals within the same country due to the epidemiological conditions mentioned above. In addition, the risk factors associated with *Eimeria* spp. included origin, sex, and extensive breeding system, consistent with the previous studies [30, 31].

The prevalence of STEs was 35.3% in the feces of all the studied cattle, consistent with the studies from Mantaro Valley (Junín Region, southern Peru), which showed 30% positivity, and Indonesia (35.7%). However, a survey conducted in 2015 showed that the Amazon region showed a 29.1% prevalence [32–34]. This data support the hypothesis that the number of cattle infected with STEs has increased in the Amazon region in recent years.

Another study indicated that the risk factors associated with STEs include the type of extensive rearing and smaller herds [35]. This might be because, in the extensive rearing system, the animals are exposed to humid areas, which are optimal survival conditions for the eggs and larvae [36]. In the Amazon region, the presence of fewer animals within a farm is closely associated with families living in poverty and linked to sanitary deficiencies regarding the deworming of livestock.

The intermittent release of eggs by *F. hepatica* is related to its age and stage and the infection and reinfection stages [37, 38], making it challenging to identify eggs using coprological tests, which decreases the sensitivity and specificity of this test. However, several authors maintained that coprological tests are feasible for identifying positive animals if more than 10 g of fecal samples is used. The presence of more than ten worms in the liver has a sensitivity and specificity of 80%–90% [39–41], respectively. As mentioned in the materials and methods section, the feces were collected between November and January (the beginning of winter), when the release of *F. hepatica* eggs rises [42]. Thus, it is highly probable that more animals tested positive for this parasite.

Based on this information, differences in the parasite prevalence rates reported in animals are due to the use of immunological or coprological diagnostic tests. The former is much more sensitive and specific than the latter (which does not allow robust identification) [16, 43]. However, in South America, coprological tests are frequently used as immunological tests are expensive and require a significantly large sample size. Logically, if we used immunological tests in this study, the prevalence would be even higher without diminishing the value of the methods used in this research.

Finally, the statistical association between *Eimeria* spp. and the presence of STEs in the same animal was also reported in investigations from India and Brazil, which showed that calves are most likely to develop coinfection [44, 45]. It is noteworthy that *Eimeria* spp. and other gastrointestinal parasites develop in the same habitat, and most of them have a direct biological cycle (without requiring an intermediate host), demonstrating the existence of both in humid pastures or without drainage [46].

## Conclusion

The high prevalence of parasites in cattle was particularly related to epidemiological factors, such as origin, drinking water sources, age, herd size, and extensive breeding. Similarly, the animals infected with STEs were also highly correlated to *Eimeria* spp. As the risk factors related to the studied parasites are not entirely clear in Peru, their identification will allow an accurate analysis of the current situation. Accordingly, control measures can be adopted in livestock areas of the Amazon region. Future studies should aim to identify other risk factors to evaluate various epidemiological mechanisms. For this, more sensitive and specific diagnostic tests should be used to identify the false-negative results that were not detected by coprological tests.

## Authors’ Contributions

HF and CM: Conception, designed, and supervised the study. MAA and DIY: Statistical analysis and data interpretation. JRP, JVC, and NLMV: Supervision, methodology, and writing - review and editing. YRB, GTS, RER, and RMLL: Laboratory analysis, data acquisition, and storage. All authors have read, reviewed, and approved the final manuscript.
